# “In sickness and in health” – how neutrophil extracellular trap (NET) works in infections, selected diseases and pregnancy

**DOI:** 10.1186/s12950-019-0222-2

**Published:** 2019-06-28

**Authors:** Paulina Niedźwiedzka-Rystwej, Weronika Repka, Beata Tokarz-Deptuła, Wiesław Deptuła

**Affiliations:** 1 Department of Immunology, Faculty of Biology, University of Szczecin, Felczaka 3c, 71-412 Szczecin, Poland; 2 Department of Microbiology, Faculty of Biology, University of Szczecin, Felczaka 3c, 71-412 Szczecin, Poland; 3 Scientific Student’s Association for Microbiology, University of Szczecin, Szczecin, Poland

**Keywords:** NET, neutrophil, immunology

## Abstract

The discovery of the NET network (neutrophil extracellular trap) has revolutionized the perception of defense mechanisms used by neutrophils in infections and non-infectious states, as this mechanism proves the complexity of the ways in which neutrophils can act in the organism. The paper describes the NET network and its participation in bacterial, viral, fungal and parasitic infections, both in a positive and a negative aspect. In addition, attention was paid to the participation of NETs in the course of autoimmune diseases, cancer, as well as its impact on pregnancy and fertility in mammals.

## Introduction

Neutrophil granulocytes (neutrophils, PMN - polymorphonuclear leukocytes) play a major role in natural immunity. They are characterized by their ability to conduct or feature phagocytosis, pinocytosis, cytotoxicity and cytolysis and form the first line of defense in antimicrobial immunity [1–5]. A new function of neutrophil granulocytes, their ability to form so-called NETs (neutrophil extracellular traps) was discovered in 2004 [6]. Microorganisms are bound outside cells in a network that can be called ‘extracellular phagocytosis’. This new feature of PMN cells has changed the understanding of their defense mechanisms as they display cidal activity not only intra- but also extracellularly.

NETs were first identified in mice and humans. Later on, the ability to form extracellular traps was confirmed in other mammals, including cattle, horses, cats, rabbits, birds, fish and invertebrates [7, 8]. More recently, the ability to form extracellular networks apart from mammals was found in eosinophils - EET (eosinophil extracellular trap) network [9], macrophages/monocytes - MET (macrophage/monocyte extracellular trap) network [10], mastocytes - MCET (mast cell extracellular trap) network [11, 12], basophils - BET (basophil extracellular trap) network [13], heterophils in birds - HET (heterophil extracellular trap) network [14], phagocytes in fish [15] (mainly in carp’s neutrophils) and hemocytes in invertebrates, in marine decapods [16] and celomocytes in worms. There is no data in literature on existence of ETs in reptiles and amphibian, but there is possibility of finding such a structure in plants. Platelets are also involved in the formation of ETs (extracellular traps). When activated, they bind to neutrophils through TLR4 receptor and facilitate network formation [17, 18]. It was shown [19], that platelets, by aggregating to NET, influence its functioning and enlarge the trap with erythrocytes and other serum factors, like von Willebrand factor, fibronectin, fibrinogen, which stabilizes the NET.

There are data suggesting that respiratory burst and formation of ROS is crucial for NET formation. It was shown that NADPH oxidase-derived ROS influence NET formation [20], but also other sources of ROS have been desribed as potentially useful for NET formation, such as singlet oxygen [21] and HOCl and HOBr [22]. Nevertheless, ROS-independent NETosis also appears and the NET formation is induced by calcium influx [23]. Moreover, chromation decondensation during NET formation may be caused by histone citrillination catalyzed by PAD4 (peptidylarginine deiminase 4) [24], but the role of histone deimination in the process of NET formation is still to be explored. The are also some studies showing that autophagy promotes formation of bacterial peptide N-formyl-Met-Leu-Phe - induced NET network [25], but the role of it is still debatable. Other factors that stimulate neutrophils into forming NETs are LPS, TNFα, IL-8, PAF, PMA [2], monosodium urate [8, 26], cholesterol [26, 27] and calcium carbonate crystals [26, 28].

NETs are formed of material from cell nucleus breakdown and neutrophil granules, including NE, MPO, cathepsin G, metalloproteinase-9, BPI protein, lactoferrin and gelatinase [6], however the core of the trap is contitiuted with chromatin with the diameter of 15-17 nm with protein domains with the diameter of 25 nm [2]. Moreover, histones from the cell nucleus (H1, H2A, H3 and H4) are 70% of the NET proteins [4]. Other constituent components include granular proteins, including proteinase 3, neutrophil serine proteases and cathelicidin (peptide LL-37) which in humans is indirectly implicated in killing microorganisms involved with NET network [3]. The peptide can extend the network life by providing protection against degradation by bacterial nucleases [3]. When a neutrophil is activated, a cascade of events occurs in effect resulting in cell breakdown. Inside the nucleus, decondensation of chromatin skeleton takes place, division into eu- and heterochromatin ceases to exist and after the rupture of the nuclear envelope stretches of DNA reach out into cytoplasm where nuclear histones and cytoplasmic granules and proteins are ‘mixed’. The cell membrane breaks down too and the whole genetic material together with the mixture of mainly proteins is transported outside the cell. As a result of NET formation, neutrophils die in an isolated death mechanism, known as NETosis [1, 2, 4].

It has been recently described that this death can occur in two forms - as lytic NETosis, known also as suicidal NETosis and non-suicidal pathway, called vital NETosis. Conventional and frequently registered suicidal NETosis starts with ligand binding to neutrophil TLR and receptors for IgG-Fc, complement and cytokines, which causes calcium ions release to the cytoplasm. High level of calcium ions in cytoplasm induces ROS and under the influence of ROS cell membrane is destroyed and substances (NE and MPO) from granules migate to nucleus, causing chromatin decondensation. This allows the release of NET but at the expense of cell life [29]. Contrary to suicidal NETosis, vital NETosis is a pathway, during which NET release occurs via blebbing of the nuclear envelope. It was reported that such patway may be stimulated by LPS and appears rapidly, even 30 minutes after stimulation, comparing to hours for suicidal NETosis [30]. This kind of NETosis has been confirmed for procesess induced by *Staphylococcus aureus* [31] and *Candida albicans* [32]. The only question raises wheather neutrophils after vital NETosis should be termed “viable”, since they lack parts of DNA.

### NETs in bacterial infections

Although there is an extensive body of reports on NET formation in bacterial infections, the way in which bacteria bind to the network strands remains not fully explained. One of the theses claims that the process relies on electrostatic interactions between negatively charged chromatin strands and positively charged surface of bacteria [18]. However, individual *Escherichia coli* or *Klebsiella pneumoniae* bacteria are thought to poorly induce neutrophil activation and NET formation [33]. *Mycobacterium bovis* Bacillus Calmette-Guérin (BCG) was used to demonstrate that only a larger amount of the bacteria can facilitate neutrophil extracellular trap release [33]. The lack of certain granules in NETs was shown to play a major role in the pathogenesis of some acute bacterial infections, since the composition of NETs translates into their efficacy [34]. A low concentration of cathepsin G in mice was found to increase their susceptibility to infection with Gram-positive bacteria which not having adequate levels of neutrophil elastase were more frequently infected with Gram-negative bacteria, including *Escherichia coli*, *Klebsiella pneumoniae* or other bacteria from the family *Enterobacteriaceae* [4]. DNA was also shown to be a NET microbicidal component and facilitate the killing of *Pseudomonas aeruginosa* [35]. The phenomenon was investigated *in vivo* in mice. After the addition of surplus Mg^2+^ ions or alkaline phosphatase, DNA had the ability to chelate ions which is pivotal for NET microbicidal activity against *Pseudomonas aeruginosa*. The same components also inhibited production of components stabilizing the bacterial membrane and proteins protecting bacteria against NET inactivation. Therefore, DNA was concluded to contribute to antibacterial properties of NET and it also may serve as a signal to elicit host-resistance stategies [35]. Some antibiotics, including clindamycin and amoxicillin, may induce NET release into extracellular environment [36]. *Neisseria gonorrhoeae* infection, even though it causes delay in phagosome formation, also induces the formation of NETs [4]. NETs play an important role in systemic infection patients [37] and can affect them in two ways. NETs can use their microbicidal ability to control infection development, catch and destroy pathogens circulating in the blood. On the other hand though, NETs can facilitate tissue and organ damage. Furthermore, NETs degraded by recombinant human DNase promote bacterial infection since mice administered with rhDNase had reduced concentration of free DNA. Unfortunately, it did not alleviate the symptoms. The same study showed that NETs was implicated in systemic tissue damage in severe sepsis [37]. The results were consistent in cystic fibrosis patients whose most effective currently available therapy is rhDNase administration. rhDNase breaks down neutrophil DNA from mucus as the rhDNase can much more quickly digest free DNA than DNA-protein complexes in NET structure [18, 38]. Although the role of NE is observed in the process, its function seems to be ambiguous. On the one hand, NE facilitates sputum dilution through histone degradation and provides access to rhDNases. On the other hand, NE release, similar to other proteolytic proteins, can damage lung tissue and enhance immune response through modulation of proinflammatory factors [18, 38].

It is worth mentioning, that NET may also be understood as a double-edge sword of innate immunity. Some bacteria were demonstrated to use their defense mechanisms that enabled them to avoid getting caught and killed by NETs [39]. One of the mechanisms is molecular mimicry whose objective is to hide molecules of antigenic bacteria to avoid immune response. A bacterial capsule is formed which contains a layer of polysaccharides similar to sialic acid of the host. The bacteria can also modify their surface through changing their charge from cation into anion. This is due to activation of *dlt* operon in bacterial genome which results in the rest of d-alanine getting bound to LTAs. Consequently, the charge is changed from negative to positive. The mechanism makes it possible to avoid immune response. Positively charged bacterial capsules repel NETs with AMPs [39]. In another defense mechanism used by pathogens to avoid getting caught in NETs, DNase is released. As it can break down DNA, the fundamental block of the network, it causes its degradation and dysfunction [35]. Although DNases are commonly released by Gram-negative bacteria, some cases of Gram-positive bacteria releasing DNases into extracellular environment were also recorded, including GAS – group A *Streptococcus* and *Streptococcus pneumoniae* [40]. *Streptococcus pneumoniae* are caught by the network of NETs and the endA gene encoding endonuclease A is expressed [6]. The presence of endA enzyme was shown to disturb the integrity of neutrophil granulocyte through its stimulation towards elastase release. Interestingly, endA release from *Streptococcus pneumoniae* in response to NET binding can be a factor of virulence of the bacteria. It is thanks to the enzyme that bacteria can spread further into the lungs and the circulatory system [40].

A study conducted on group A streptococcus showed that the group uses another enzyme, DNase Sda1, to escape NETs. It improves bacterial survival and their virulence both *in vivo* and *in vitro* [41]. Another bacterial defense mechanism is to trigger an exaggerated immune response as bacteria come into frequent contact with neutrophils, thus enhancing their proinflammatory activity. Group A strep (GAS) bacteria [42] were demonstrated to produce M1 protein, a key component for their virulence. M1 causes NET formation and release while at the same time allows proteins from the network to escape degradation. Escape from neutrophil extracellular traps may also involve blocking the network from forming in neutrophils in the first place [17]. In *Streptococcus pyogenes* bacteria that produce SpyCEP protease, IL-8 is inactivated and GAS-stimulated NET formation rate is reduced which helps pathogens avoid extracellular killing [17].

### NETs in viral infections

NETs can bind and immobilize viruses through electrostatic interactions between virus molecules and the chromatin skeleton of the network [43–46]. Additionally, histones present in NETs, rich in positively charged amino acids, can bind virus capsids negatively charged [43]. H3 and H4 histones were shown to aggregate in their structure type A influenza virus while H1 binds some noroviruses [43]. Recent research showed that some NET components, including myeloperoxidase, cathelicidin and α-defensins, display strong antiviral properties. In particular, α-defensins show their biocidal activity both in enveloped and non-enveloped viruses [43]. The human immunodeficiency virus HIV-1 was found to induce suicidal NETosis recognized by neutrophils mainly through TLR7 and TLR8 (Toll-like Receptors) [46]. The engagement of the receptors causes a cascade of events resulting in the production of ROS, followed by NET formation and destruction of the virus [46]. Interestingly, HIV-1 has developed defense mechanisms against NET. HIV-1 increases IL-10 production by DCs, holding against ROS generation and thus inhibiting NET release [46]. Influenza type A virus is another virus that can induce NETosis. The virus is associated with ARDS (acute respiratory distress syndrome) which causes NET formation following activation of epithelial cells of the lungs [47]. Other inducers of NETosis include oxidoreductases active in infection, super-oxidized ions and H_2_O_2,_ from epithelial cells [43]. Hantaan virus also generates NET release and the cytotoxic properties of the network may exacerbate pathological changes of the organism induced by the virus infection [48]. NET histones generated in Hantaan virus infection facilitate thrombin generation and intravascular coagulation. They increase the permeability of vascular endothelial cells and induce production of antibodies thus exacerbating the pathological condition caused by the virus [48]. NS1 (non-structural protein 1) is the key protein that activates NETosis in Dengue virus infection [49]. The protein activates cells that have not been infected, including endothelial cells, via TLR4. As a result, endothelial cells stimulate neutrophils to form NETs. IL-8 produced by activated endothelial cells facilitates NET.

### NETs in fungal infections

NETs can inhibit the growth and development of *Candida albicans*, *Aspergillus nidulans*, *Cryptococcus neoformans, Aspergilus fumicatus*, *Candida glabrata* and *Cryptococcus gattii* [50, 51] although the mechanism behind this activity has not been fully explained. Calprotectin [50] was found to be the crucial protein involved in NET degradation of fungi. The fungicidal activity of calprotectin was found in a study on *Candida albicans*. Chelation of Zn^2+^ or Mn^2+^ ions, which are of importance for fungi, reduced their growth [50]. The same study showed that increased concentrations of the ions inhibit NET activity [50]. Furthermore, *in vitro* observations showed that when calprotectin is exhausted, NET inhibitory activity comes to an end [50]. Mice with calprotectin deficit are not able to prevent *C. albicans* infection [50]. It was also discussed that neutrophils sense microbe size, and that NET may be the mechanism of response to *Candida albicans* hyphae, but not to for yeast form [33]. Intriguingly, Branzk et al. [33] claim that NET is not forming in response to small yeast or single bacteria, whereas in different studies [52], it is claimed that hyphae, as well as yeast form can by caught by NET. The hyphae of *Candida albicans* after coming into contact with activated neutrophils cause histone degradation which affects further chromatin decondensation during NET release [33]. The study by Branzk et al. also shows [33] that selective death of fungus cells in the process of NETosis does not depend on enzymatic activity of the organisms as high temperature-inactivated *C. albicans* strands initiate NET release to the same extend as alive strands.

Similarly, in *Aspergillus fumigatus* infection, NETs are released in response to fungus strands or large agglomerates of conidia. However, NETs are not released when only individual conidia exist in extracellular environment [33]. Bruns et al. [52] demonstrated that strands of *A. fumigatus* are stronger inducers of NET formation than conidia. This finding can be linked to the presence of RodA hydrophobin. The protein occurs only on conidium surface which makes it immunologically indifferent and thus inhibits NET formation. This finding is consistent with another study [38] that showed that some components of fungal cell walls can trigger NET formation. The study demonstrated that while glucans initiate NET release in a ROS-regulated pathway mannans rely on a ROS-independent route [38].

### NETs in parasitic infections

NETs were identified in infections with *Leishmania*, *Plasmodium*, *Eimeria*, *Toxoplasma* and *Strongyloides* parasites [53–57]. Contact with these protozoa is possible only through a vector, e.g. a mosquito or a tick. A pathogen is inoculated into blood through its organism [33]. NETs may be necessary to control parasites and modulate immune response through their constituent components [54]. Both promastigote and amastigote forms of parasites from the genus *Leishmania (L.)* are able to initiate NET formation, as was demonstrated by the presence of DNA strands in extracellular environment [54]. LPG found on the surface of promastigota cell form was shown to induce neutrophils into NET release. This is due to the fact that the negative charge on promastigote surface that regulates its binding to NETs can at least in part be also regulated by LPG presence [54]. The promastigote form of *L. donovani* is capable of NET induction although *L. donovani* and *L. major* promastigotes are resistant to NET-regulated killing [54].

NETs and histones present in the network are toxic for promastigotes [54]. A study conducted to identify susceptibility of successive species of *Leishmania (L.) sp.* to histone activity showed that promastigote forms of *L. mexicana*, *L. brasiliensis*, *L. major* and *L. amazonensis* are susceptible. Their protein-induced death mechanism remains to be explained. The amastigote forms of *L. mexicana* and *L. amanozonenis* are resistant to the toxic activity of histones. Infection with *Plasmodium,* another protozoa, can stimulate NET formation. It was reported that NET is present in the blood of patients infected with *Plasmodium falciparum, Plasmodium vivax* and *Plasmodium malariae* [56].

A role played by NETs was observed also in infections with *Eimeria bovis* [55]. The parasite was demonstrated to induce NET formation via calf neutrophils and to trap and bind the sporozoites. Infections with *Toxoplasma gondii* in humans and mice stimulates NET formation [47]. Bonne-Année et al. [53] demonstrated that NETs are also induced by *Strongyloides stercoralis*, a parasitic roundworm common in humans, primates and dogs. The study showed that although the network of neutrophil extracellular traps binds the parasites *in vitro*, it does not kill them [53].

### NETs in autoimmune diseases

NET disfunction can result in infection or development of many non-infectious diseases in humans and in other mammals, including mice and cattle [58]. The accumulation of NETs released during infection or pathological condition can injure adjacent tissue. Irregular removal of NETs from the organism causes pathological conditions in the macroorganism. Endogenous DNase may be implicated in the process since their reduced levels disturb successful NET degradation [59]. On the other hand, destruction of DNA skeleton, a NET element, releases antibacterial proteins which can cause massive damage of adjacent tissues [59]. For example, myeloperoxidase released in this way inactivates metalloprotease inhibitors. Consequently, pathological changes caused by the enzymes can occur. Another example is provided by neutrophil elastase release from NET skeleton which can damage barriers formed by the epithelium, e.g. in the lungs [59].

One of the negative effects of NET presence in extracellular environment is that NETs are involved in autoimmune diseases. During NET formation, citrullinated histones and antigens are released into extracellular environment and are presented to immunocompetent cells which in response start producing ANCAs and ANAs [60, 61]. The antibodies usually act against myeloperoxidase, proteinase 3 and neutrophil elastase. As a result, neutrophils get activated and NETs are released [15]. NETs contain autoantigens, including myeloperoxidase and proteinase 3, which become the target of successive antibodies. Thus, an autoactivation loop is created which is the underlying component of autoimmune disease development. Abnormal NET formation, issues with network degradation and lack of equilibrium between the two are the factors initiating or facilitating autoimmune diseases, including systemic lupus erythematosus, rheumatoid arthritis and vasculitis [59–63]. Antibodies are produced by B lymphocytes against nuclear antigens, e.g. DNA, histones or RNPs and against ANCAs, in SLE [59]. That leads to a typical feature of the disease, i.e. formation of immune complexes and long-term activation of pDCs [59]. Abnormal removal of cells undergoing apoptosis was recorded in which the release of intracellular autoantigens caused constant production of autoantibodies which is considered to be the key mechanism of the pathogenesis of the disease [59]. The mechanism of its pathophysiology has a role for genes, hormones, environment and abnormal lymphocytes which may affect development of the disease [59]. Having described cell death in NETosis, NETs became the successive and likely sources of autoantigens produced in the disease. Thus, the phenomenon was redefined as one cause of the disease [59]. A link between autoantigen-releasing cells, including plasmacytoid dendritic cells, B lymphocytes, antibodies, and NETs was demonstrated [64]. Neutrophils primed with type I interferons and exposed to autoantibodies produced NETs [51]. NET components can then activate B lymphocytes via their specific BCRs and TLR-9. Then follows production of autoantibodies against their own DNA and cathelicidin (LL-37) which occur in large amounts in SLE patients [65]. The antibodies can bind DNA and cathelicidin (LL-37) from NETs and form autoantibody-DNA/LL-37 complexes which are recognized by B cells and pDCs and then “sustain” autoimmune infections [65]. A correlation between insufficient NET degradation and development of the disease was found in SLE [59]. The serum of SLE patients had reduced ability to degrade NETs. Accordingly, patients could be divided into non-degraders and degraders with higher NET degradation activity [59].

Another study on SLE [61] showed that the ability to degrade NETs is correlated with its activity. Accordingly, observations showed that increased ability to NET degradation occurred in remission and decreased ability to do so happened in relapses. Moreover, patients with worsened condition had increased levels of NET antibodies. Undegraded NET in SLE was demonstrated to activate the complement system and C1q, a component of the system degrades NETs. The opsonization of NETs or direct inhibition of DNase I are the hypothesized origins of the phenomenon [61].

Another autoimmune disease that involves NETs is rheumatoid arthritis (RA). The serum of RA patients contains a range of autoantibodies, including antifilagrine antibodies, anti-creatinine antibodies and antiperinuclear factors [4, 63]. The serum can be used to obtain fundamental diagnostic parameters, including rheumatoid factor, ACPAs and importantly anti-citrullinated protein antibodies [63]. Proteins that citrullinate during NET formation have increased antigenicity which generates production of their antibodies [53]. Khandpur et al. showed [63] that there is a correlation between NETs and ACPA presence and concentration in the blood. RA serum antibodies were observed to react with NET histones [63]. Therefore, NETs are speculated to display immune properties which can trigger a vicious circle of immune reactions. ACPA-binding NETs in the joint lead to the neutrophil Fcγ receptor binding to immune complex which further facilitates NET release [63]. A correlation was found between NET formation and systemic inflammatory markers [63]. Khandpur et al. [63] showed that neutrophils taken from RA patients are more prone to form NETs than those from healthy individuals. IL-17 and TNF-α also derived from the serum are able to induce NETs in RA neutrophils.

### NETs in cancer

Anticancer effect of neutrophils is known for several years [66]. Following direct interactions, neutrophils induce migration of cancer cells, simulate their growth via metalloproteinases released from the extracellular matrix and facilitate angiogenesis [66]. Conversely, activated neutrophils exert cytotoxic effect on cancer cells by the release of ROS and defensins [66]. G-CSF that is produced by most types of cancer cells activates neutrophils and stimulates them to NET formation [66]. Some NET components, including myeloperoxidase, proteinases and histones, can have a cytotoxic impact on cancer cells and inhibit cancer growth [66]. NETs are suggested to bind cancer cells, thus blocking their further proliferation in the organism [66].

The alternative view on the role of NETs says that through the activation of its protein components the network can facilitate extravasation and metastasis [67]. NETs are thought to ultimately stick to metastasis cells. Through the recruitment of platelets, NETs can protect cancer cells and inhibit immune response [67]. NETs were also shown to directly trigger malignant transformation of cancer [67]. In a study conducted on cancer mice, infection-induced NETs could facilitate metastasis through trapping cancer cells in the circulatory system and facilitate tumor relapse after its surgical resection in patients with metastatic colorectal cancer [68].

Recent studies of Albrengues et al. [69], it was reported that sustained lung inflammation induced the NET formation and awaken dormant cancer. It was shown [I], that two components of NET – NE and MMP9 caused laminin cleavage, which induced proliferation of dormant cancer cells. Moreover, antibodies against NET-remodeled laminin prevented awaking of dormant cells, giving the first step of a new pathway to prolong the survival of cancer patients [69].

### NETs in pregnancy and fertility

The negative effect of NETs was observed in human fetal development, e.g. in preeclampsia [70, 71]. In an *in vitro* study on CD11b expression, fragments of placenta were able to induce neutrophils into the formation of NETs [70, 71]. Contrary to healthy placenta, the placenta of preeclampsia women had NETs occurring in close contact with trophoblasts [70, 71]. NETs are thus hypothesized to take the space inside trophoblast villi, to reduce vascular blood flow and to finally cause fetal hypoxia [70]. Another mechanism that can result in NET-assisted fetal hypoxia is NETosis facilitated by activated vascular endothelial cells [70]. which eventually destroys endothelial cells of the mother [70, 71]. It is unknown at this point whether NETs trigger pathological changes or the network is caused by some type of deficiency in the placenta [70, 71]. Analysis of levels of free DNA circulating in the mother and the fetus in preeclampsia showed that NETs must have been its origin. The growth of cfDNA (cell-free DNA) inside the fetus can suggest a pathological condition of the placenta [70, 71]. However, a high level of maternal free DNA suggest that its origin lies in NET generation [70, 71]. Research showed that increase of fetal cfDNA levels occurs in early pregnancy, prior to preeclampsia symptoms. However, increased maternal free DNA occurs only once the symptoms develop [64]. Since ROSs are necessary for NET formation and NET release into extracellular environment, some researchers speculate that NETs can contribute to miscarriages caused by maternal antiphospholipid antibodies [71]. Hahn et al. [71] contend that the presence of NETs can be cytotoxic for adjacent tissues, which can have an adverse effect on the fetus.

NETs in animals, mainly mares, can constitute a distinctive feature of individuals with reduced fertility [72]. The reason behind it was thought to be the ability of neutrophils to bind the spermatozoo and form widespread agglomerates which reduces sperm motility and inhibits its ability to fertilize the egg. Following NET discovery, the ability of sperm to form sperm agglomerates using NETs was investigated [72]. Spermatozoids were shown to activate neutrophils to NET formation and release in a way similar to that in which bacteria activate cells [72]. Since SP (seminal plasma) contains proteins with endonuclease capabilities, similar to DNases - e.g. DNase I, it increases sperm fertility potential in cattle, horses, and pigs through their release from NET agglomerates [72].

## Conclusions

NET formation has focused the attention of many scientists. It seems to be not only a very effective defense mechanism used by immune system cells (neutrophils) but also a structure that inevitably requires a “victim” i.e. NETosis, or cell death. NETs can act like a double-edged sword. The positive effects of NETs in pathogenic infections (bacteria, viruses, fungi and parasites) are balanced by their negative effect on the health of mammals, including humans. NETs are observed in non-infectious, including autoimmune diseases, cancer, fertility disorders in humans and animals. More recent reports implicate the role of NET formation in Alzheimer’s disease and sterile inflammation.

## Data Availability

Not applicable

## Abbreviations

ACPA: Anti-citrullinated protein antibodies
AMP: Antimicrobial peptides
ANAs: Anti-nuclear antibodies
ANCAs: Anti-neutrophil cytoplasmic antibodies
BET: Basophil extracellular trap
EET: Eosinophil extracellular trap
G-CSF: Granulocyte-colony stimulating factor
HET: Heterophil extracellular trap
LPG: Lipophosphoglycan
LPS: Lipopolysaccharyde
LTA: Lipoteichoic acid
MCET: Mast cell extracellular trap
MET: Macrophage/monocyte extracellular trap
MMP9: Metalloproteinase 9
MPO: Myeloperoxidase
NE: Neutrophil elastase
NET: Neutrophil extracellular trap
PAF: Platelet-activating factor
PMA: Phorbol myristate acetate
PMN: Polymorphonuclear leukocytes
RA: Rheumatoid arthritis
RNPs: Ribonucleoproteins
ROS: Reactive oxygen species
SLE: Systemic lupus erythematosus
TNFα: Tumor necrosis factor
